# Lung Function and Relevant Clinical Factors in Very Low Birth Weight Preterm Infants with Chronic Lung Disease: An Observational Study

**DOI:** 10.1155/2019/5824180

**Published:** 2019-08-05

**Authors:** I-Ling Chen, Hsiu-Lin Chen

**Affiliations:** ^1^School of Life Sciences, University of Nottingham, Nottingham NG7 2RD, UK; ^2^Department of Pediatrics, Kaohsiung Medical University Hospital, No. 100, Tzyou 1st Road, Sanming District, Kaohsiung City, Taiwan; ^3^Department of Respiratory Therapy, College of Medicine, Kaohsiung Medical University, No. 100, Shih-Chuan 1st Road, Sanming District, Kaohsiung City, Taiwan

## Abstract

**Background:**

Chronic lung disease (CLD), most commonly seen in premature infants who required mechanical ventilation, is associated with functional consequences on lungs and respiratory morbidity. This study aimed to evaluate the lung function of premature infants before discharge and their relevant factors related to the lung function.

**Methods:**

Very low birth weight (VLBW) preterm infants, who required respiratory support soon after birth and were admitted to a hospital in Taiwan, were enrolled. Infants with a need for supplemental oxygen or positive-pressure ventilation support at the postmenstrual age (PMA) of 36 weeks were diagnosed with CLD. Lung function was examined once using EXHALYZER® D before infants were ready for discharge.

**Results:**

Forty-five VLBW preterm infants received the lung function test before discharge, 27 of whom were diagnosed with CLD. The gestational age (*p*=0.001) and birth weight (*p* < 0.001) were smaller in the CLD group than in the no-CLD group. Furthermore, infants with CLD required a longer duration of respiratory support (*p* < 0.001). The postnatal age and PMA were higher and body size was bigger in infants with CLD on lung function measurement. However, lung function was comparable between the groups. The functional residual capacity and tidal volume were associated with body size upon measuring lung function among all VLBW premature infants. FRC was positively correlated with the body length on measuring lung function in those with CLD.

**Conclusion:**

In our study, we showed FRC was positively related to the PMA and body length and tidal volume was positively correlated with the body weight and length on lung function measurement in VLBW preterm infants before discharge. Moreover, FRC was positively correlated with the body length on measuring lung function in those with CLD. The lung volume, ventilation, and respiratory mechanics on discharge were comparable between CLD and no-CLD groups.

## 1. Introduction

Survival of low birth weight preterm infants is improving because of advances in neonatal care; however, the complications of survived infants might increase simultaneously. Chronic lung disease (CLD) of prematurity, also known as bronchopulmonary dysplasia (BPD), is commonly found in preterm infants who require mechanical ventilation and/or oxygen support. CLD has been defined as persistent oxygen dependency up to 28 days of life or a need for supplemental oxygen at the postmenstrual age (PMA) of 36 weeks [1–3]. The average incidence of CLD is 43% for preterm infants with <28 weeks of gestational age over the previous few decades [4] and 34.9% for very low birth weight (VLBW) preterm infants in Taiwan [5]. CLD results from a complex process by which pulmonary vascular growth and alveolarization are interrupted, leading to ineffective gas exchange [2, 6]. In addition to prolonged respiratory support, both antenatal factors [7] (e.g., glucocorticoids, chorioamnionitis, and genetic susceptibility) and postnatal factors [2, 3] (e.g., hyperoxia, oxidative stress, and inflammation) contribute to CLD development.

Abnormal lung development in infants with CLD has been reported to have deviations in lung function. Compared with healthy preterm infants, those with CLD had reduced functional residual capacity (FRC) depending on severity of the disease [8]. Furthermore, CLD might induce long-term effects on lung function. CLD survivors with very low birth weight had lower forced vital capacity (FVC) [9], forced expiratory flow at 50% of vital capacity (FEF_50_) [9], forced expiratory volume in one second (FEV_1_) [9, 10], and forced midexpiratory flow (FEF_25–75_) [10] at school age. Moreover, a diminished FEV1/FVC of CLD survivors was observed in late adolescence [11].

The application of pulmonary function testing in infancy and childhood has been recommended as a clinical training and research priority [1]; however, it is challenging to measure lung function in infants. Existing methods, including the rapid thoracic compression technique and whole-body plethysmography, had several restrictions in capacity and require sedation [12, 13]. We used the commercial lung function test device, EXHALYZER® D. Multiple-breath washout and ultrasonic transit-time measurements were taken by EXHALYZER® D to assess lung volume and ventilation distribution in spontaneously breathing and unsedated infants. EXHALYZER® D has been validated as a technique to obtain lung function with sufficient accuracy and reproducibility without disturbing the breathing pattern [13].

The aim of this study is to assess the lung function of premature infants before discharge from hospital and to determine if lung function correlated with clinical variables in CLD of prematurity.

## 2. Methods

### 2.1. Study Population

This was a clinical observational study. Participants were recruited from the Neonatal Intensive Care Unit of Kaohsiung Medical University Hospital in Taiwan between 2011 and 2013. Infants with very low birth weight (≤1500 g) were eligible for the study if they met the following criteria: [1] gestational age less than 37 weeks and [2] and needed respiratory support soon after birth. The criteria for exclusion were those with any major birth defects/chromosomal abnormalities or those without informed consent. The clinical determinants (i.e., gestational age, birth weight, gender, antenatal use of steroids, severity of respiratory distress syndrome (RDS) determined by chest X-ray on admission based on radiographic thorax findings according to Giedion grades I–IV [14], and patent ductus arteriosus (PDA)), detailed medication, and mode and duration of respiratory support were recorded regularly during hospital stay into a database. Participants were further categorized into two groups based on their diagnosis of CLD, which was defined as a need for supplemental oxygen or positive-pressure ventilatory support (including invasive positive ventilation and nasal continuous positive airway pressure (CPAP)) at the PMA of 36 weeks. PMA is used to describe the age of infants in weeks on measuring lung function since the first day of the last menstrual period, that is, gestational age plus chronological age. Body weight *z*-scores and body length *z*-scores were calculated based on the 2013 Fenton growth chart for preterm infants [15]. Preterm infants in our hospital were supported with gentle ventilation since birth. Respiratory support was provided based on early CPAP (5 cmH_2_O through mask or short binasal prongs) after birth. For intubated infants, nasal CPAP was applied after extubation followed by room-air CPAP. For infants without intubation, we started weaning while they were under room-air CPAP. Subsequently, cyclic use of NCPAP with room air was used as a process of weaning from nasal CPAP. Duration of room air use during the weaning course was increased gradually along with infants' condition improvement. We did not use supplemental oxygen while weaning from nasal CPAP.

The experimental protocol was approved by the Institutional Review Board (IRB) of Kaohsiung Medical University Hospital (the IRB number is KMUH-IRB-990282), and written informed consent was obtained from all infants' parents.

### 2.2. Lung Function

Lung function tests were performed once, prior to discharge from the hospital, using EXHALYZER® D (ECO MEDICS AG, Duernten, Switzerland). EXHALYZER® D is a lung function testing system, fully compliant with the ATS/ERS recommendations, using multiple-breath washout and ultrasonic transit-time measurements. Infants were determined to be in clinically stable condition before administering lung function testing, which was within a week of hospital discharge. Lung function testing was performed via an infant face mask during unsedated sleep in the supine position. Pulse oximetry and oxygen saturation were monitored using a pulse oximeter during lung function measurement. None of the studied infants were on respiratory support at the time of examining lung function. The following parameters were recorded: functional residual capacity (FRC), tidal volume (TV), minute volume (MV), respiratory rate (RR), total inspiratory time (*T*_I_), total expiratory time (*T*_E_), and time to peak tidal expiratory flow (*T*_PTEF_). Ventilation inhomogeneity, indicated by the lung clearance index (LCI) and first and second moment ratios, was measured using the multiple-breath washout technique with 4% SF_6_ during normal tidal breathing.

### 2.3. Statistical Analysis

Descriptive data of studied infants' clinical variables are presented as mean ± standard deviation (SD). Differences in frequency distribution of categorical variables between studied infants with and without CLD were evaluated by the chi-square test. Meanwhile, Mann–Whitney's *U* test was used for analyzing numerical data. To assess association between clinical variables (i.e., gestational age, birth weight, PMA, RDS grade, PDA, total days of respiratory support, body weight, and body length on checking lung function) and parameters of lung function, Pearson's correlation test and linear regression were performed. Missing measurements were not included in the analyses. All analyses were performed with the Statistical Package for Social Sciences software (SPSS Inc, Chicago, IL, USA) for Windows version 24. Differences or associations were considered statically significant when *p* values were <0.05.

## 3. Results

### 3.1. Clinical Characteristics and Lung Function Parameters of All Participants

Of the 61 premature infants who were assessed, 45 were eligible for inclusion in the study including 25 males and 20 females with a mean gestational age of 28.16 ± 2.42 weeks and a mean birth weight of 1048 ± 252 g. Studied infants underwent lung function testing once before being discharged from the hospital, and their PMA on checking lung function was 39.94 ± 2.85 weeks.

### 3.2. Comparison of Clinical Variables and Lung Function Parameters in the Studied Infants with and without CLD

Twenty-seven studied infants (60%) were diagnosed with CLD, satisfying the following diagnostic criterion: a need for supplemental oxygen or positive-pressure ventilatory support at the PMA of 36 weeks. The gestational age (27.19 ± 1.98 weeks vs. 29.61 ± 2.33 weeks; *p*=0.001) and birth weight (0.92 ± 0.21 kg vs. 1.24 ± 0.16 kg; *p* < 0.001) of infants with CLD were lower than those of infants without CLD (Table 1). Moreover, the duration of respiratory support (72.15 ± 21.79 days vs. 29.61 ± 16.82 days; *p* < 0.001) and intubation time (22.69 ± 26.8 days vs. 2.17 ± 4.71 days; *p*=0.003) were longer in the CLD group (Table 1). Approximately 75% of the infants with CLD had PDA, which was significantly higher than that of infants without CLD (*p*=0.005; Table 1). No significant difference was observed in gender, severity of RDS, or medication use including prenatal steroids, surfactants, and aminophylline (Table 1).

The results of the lung function tests by multiple-breath washout are shown in Table 2. The postnatal age (93.30 ± 20.14 days vs. 55.39 ± 20.16 days; *p* < 0.001) and PMA (41.33 ± 2.21 weeks vs. 37.86 ± 2.42 weeks; *p* < 0.001) were higher in infants with CLD than without CLD on measuring lung function. Furthermore, infants with CLD had significantly higher body weight (2.73 ± 0.54 kg vs. 2.47 ± 0.61 kg; *p*=0.01) but not body length upon lung function measurement. Infants with CLD also had significantly lower body weight *z*-scores corresponding to PMA. All parameters of lung function were comparable between groups.

### 3.3. Correlation between Clinical Characteristics and Lung Function Parameters

**Figure 1:**
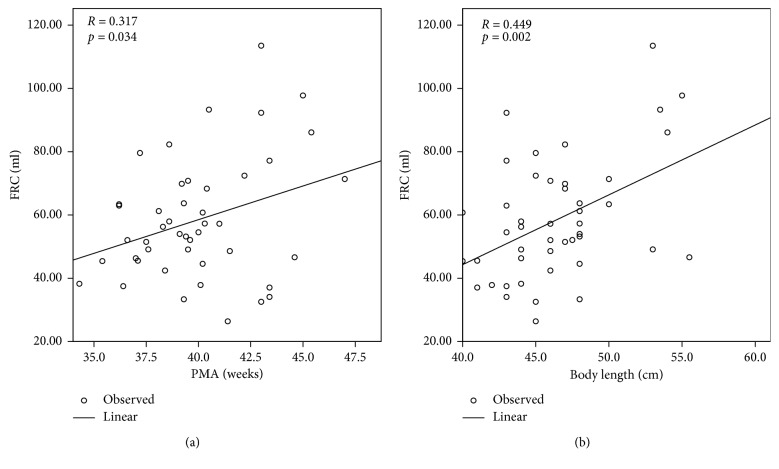
Linear regression between FRC and clinical variables in all studied infants. (a) Linear regression between FRC (ml) and PMA when measuring lung function. (b) Linear regression between FRC (ml) and body length (cm) when measuring lung function. The FRC (ml) was positively related to the PMA (*p*=0.034) and length (*p*=0.002). FRC: functional residual capacity; PMA: postmenstrual age.

**Figure 2:**
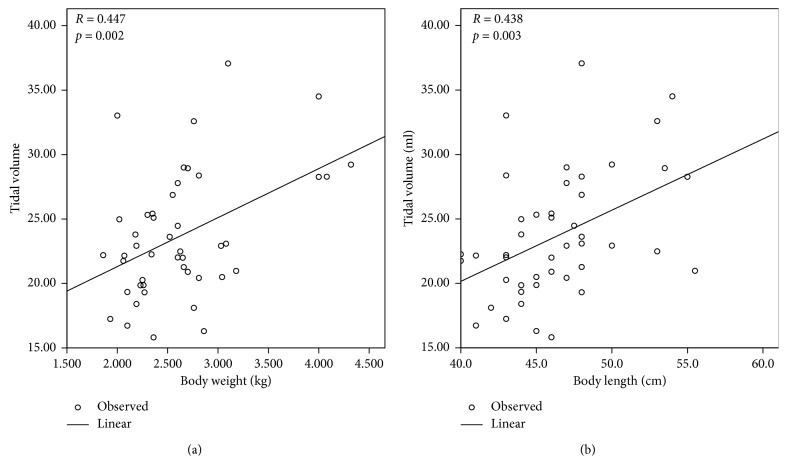
Linear regression between tidal volume and clinical variables in all studied infants. (a) Linear regression between tidal volume (ml) and body weight (kg) when measuring lung function. (b) Linear regression between tidal volume (ml) and body length (cm) when measuring lung function. Tidal volume (ml) was positively related to body weight (*p*=0.002) and length (*p*=0.003).

**Figure 3:**
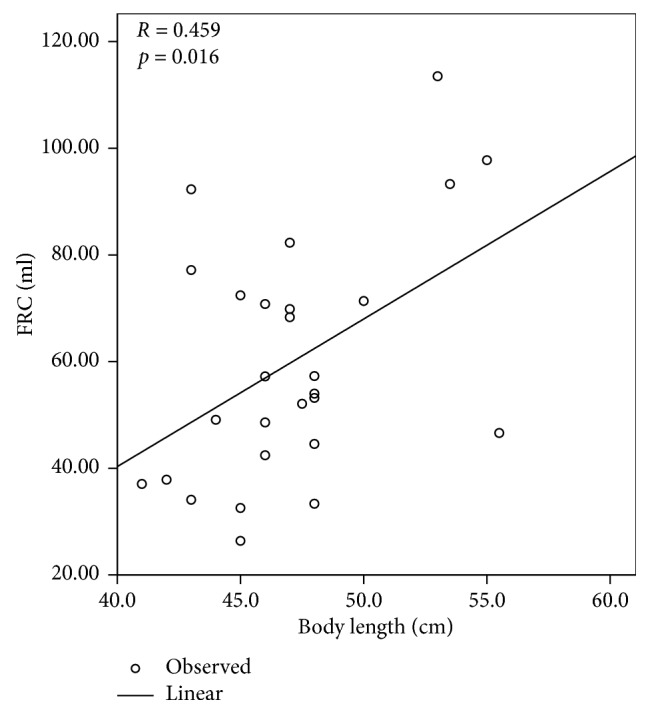
Linear regression between FRC (ml) and body length (cm) in infants with CLD when measuring lung function (*p*=0.016).

To investigate the potential factors of lung function, we performed correlation and linear regression analysis between clinical variables and parameters of lung function. We initially examined all studied infants and found the FRC (ml) was positively related to the PMA (*p*=0.034) and body length (*p*=0.002) when measuring lung function (Figure 1). Similarly, tidal volume was positively correlated with the body weight (*p*=0.002) and length (*p*=0.003) (Figure 2). We further assessed correlation in infants with CLD and found FRC (ml) was positively correlated with the body length (*p*=0.016) on measuring lung function (Figure 3). The other parameters of lung function of infants with CLD were not influenced by clinical variables (data not shown).

## 4. Discussion

In this study, we demonstrated VLBW preterm infants with CLD had significantly lower gestational age and birth weight compared to those without CLD. Lung function testing was performed once before discharge, and those VLBW preterm infants with CLD had higher postnatal age and PMA on measurement. Lung volume and ventilation inhomogeneity in very preterm infants with CLD were comparable to those in infants without CLD before discharge. FRC and tidal volume were affected by body size in VLBW preterm infants, and FRC was positively correlated with body length in those with CLD. No significant difference was observed in gender, severity of RDS, or medication use (including prenatal steroids, surfactants, and aminophylline) between preterm infants with and without CLD. Aminophylline was used exclusively in our institution because we did not purchase caffeine at the time of conducting this study. The efficacies of aminophylline and caffeine were comparable in several aspects including apnoea prevention and hospital stay in prematurity [16, 17], although aminophylline has a narrower therapeutic index.

CLD is one of the most common complications in preterm infants that results from dysmorphic growth and interruption in lung development, thereby leading to possible long-term respiratory morbidity and abnormalities in lung function. Several studies have addressed the relationship between the history of CLD and diminished lung function in childhood up to adolescence [9–11, 18]. However, it is not a common practice to measure lung function in preterm infants with CLD before discharge in our hospital. Here, we have shown that it is feasible to conduct lung function testing with a noninvasive device once before discharge to assess the respiratory condition and evaluate relevant factors to their lung function.

Approximately 40% of extremely low gestational age infants had pulmonary deterioration in the first two postnatal weeks, and 50% of these were diagnosed with CLD [19]. Indeed, we found VLBW preterm infants with CLD to have a significantly lower gestational age compared to those without CLD, which is consistent with another regional prospective cohort study [20] and also reflects the severity of disease [21]. This study also observed that VLBW preterm infants with CLD had lower birth weight, which corroborates previous evidence [20]. These findings indicate that developmental immaturity may be related to susceptibility to, and the severity of, CLD.

FRC, which can reflect growth and development of the lung, is commonly used to monitor lung disease and evaluate treatment response in infancy [22]. Studies have demonstrated a reduction in FRC in both healthy preterm infants [23] and infants with CLD [8, 24]. Elevation of FRC, residual volume (RV), and RV/TV were more pronounced in CLD patients with recurrent wheeze [25]. In contrast, Hulskamp et al. reported comparable FRC between groups with and without CLD [26], which is consistent with our findings. The differences in results between studies might be caused by several factors, including measurement devices, sedation status, and breathing patterns of the infants studied. In this study, FRC was positively associated with PMA and body length in all studied VLBW preterm infants. Furthermore, similar correlation was found between FRC and body length in infants with CLD. We consider that the lung function is related to the maturity of the lungs. PMA of preterm infants could reflect maturity of the lungs and somatic growth, and body length is known to be the major determinant of FRC [27], as confirmed in this study.

RDS is caused by inadequate levels of the surfactant in the alveolus [28], while pulmonary flow and remodeling are augmented in PDA due to a systemic-to-pulmonary shunt [29]. Both diseases increase the need for respiratory support to maintain ventilation; therefore, PDA and RDS are considered leading causes of subsequent CLD in preterm infants. Previous studies have reported that the presence of RDS [5] and PDA [30] was related to CLD development; however, we found that the rate of PDA, but not RDS severity, was significantly higher in VLBW preterm infants with CLD. The rate of surfactant use, one of the common treatments for RDS, was also comparable between groups in our study, which contrasted with one previous study in which there was more surfactant use in the CLD group [20].

Administration of steroids prenatally is used to reduce the risk of preterm delivery and the severity of RDS and might also improve responses to surfactant treatment in infants, which is attributed to elevation of structural maturity [31]. Prenatal steroid therapy is related to increasing FRC in preterm infants at 36 hours of age [22] and lower tidal volume to body weight (TV/kg) in extremely preterm infants at discharge or term age [20]. This highlights the potential role of prenatal steroids in respiratory mechanics. There was no significant difference in prenatal steroid use between CLD and no-CLD groups in this study. We could not draw conclusions as to whether it caused the comparable FRC and TV/kg in our study because there might be several factors influencing lung development in preterm infants, especially the time aspect (i.e., maturation) with PMA.

The time to peak tidal expiratory flow as a proportion of expiratory time (*T*_PTEF_/*T*_E_ ratio) is used to evaluate alterations in expiration, and a reduced ratio demonstrates airway obstruction and increased susceptibility to wheezing-associated diseases [32]. Healthy late-preterm infants born at 33–36 weeks had lower *T*_PTEF_/*T*_E_ and compliance compared to term infants [33]. Moreover, extremely preterm infants with CLD had lower *T*_PTEF_/*T*_E_ compared to those without CLD [20] and reduced *T*_PTEF_/*T*_E_ reflected the increasing severity of CLD [34]. In contrast, we did not find any significant difference in *T*_PTEF_/*T*_E_ between CLD and no-CLD groups. This might be due to the participants we recruited, whose degree of airway obstruction was not severe enough to discriminate CLD.

Indices of ventilation inhomogeneity (LCI and moment ratios) were derived from multiple-breath washout of an inert tracer gas (in this case, 4% SF_6_) from lungs during relaxed tidal breathing. LCI has been used to assess small airway diseases with good reproducibility and accuracy in children [35]. Elevation of either LCI or moment ratios was observed in infants with reduced ventilation efficiency [36]. We did not notice any significant difference in ventilation inhomogeneity between the CLD and no-CLD groups, which is consistent with previous findings which showed no discrimination when assessing CLD in either infancy [37] or childhood [38]. No clinical variables were related to the indices of ventilation inhomogeneity in our study, although LCI was associated with duration of supplemental oxygen reported by Hulskamp et al. [37]. Development of CLD occurred in the lung periphery and involved arrested alveolar growth and dysmorphic arteries; however, no changes in LCI or moment ratios suggest that CLD affected only the structural homogeneity but not ventilation distribution.

Although infants with CLD had lower body weight and body weight *z*-scores (which present standard deviations from mean body weight of infants at same PMA), all lung function parameters were comparable between the CLD and no-CLD groups. We have further classified the infants with no, mild, and severe CLD according to their oxygen dependency at 36 weeks of PMA [1] and compared their lung function. Again, there was no significant difference in lung function between groups (data not shown). Since the definition of CLD used in this study is simply based on the requirement of oxygen but does not consider ventilation (i.e., CO_2_ washout), our findings suggest that the current definition of CLD is not able to reflect the lung maturity; thus, a more comprehensive definition may be required. A study reported that functional lung impairment of CLD was of the same nature as healthy preterm infants which proposed the similar mechanisms involved in pathological lung development of CLD and uncomplicated premature infants [8]. This may also explain the comparable lung function between CLD and no-CLD groups in this study. The current findings may also provide some useful information for the clinical management of preterm infants. Since lung function was comparable between VLBW preterm infants with and without CLD, it may not be necessary to perform lung function tests on preterm infants on discharge, especially in those hospitals having limited budgets. Hospitals which are not able to perform lung function tests regularly could consider raising VLBW preterm infants to bigger sizes and greater maturity to improve their lung function in view of the positive correlation between FRC and body size. Certainly, a larger sample size may be needed for future studies.

## 5. Conclusions

We have shown that it is feasible to measure lung function of VLBW preterm infants in this study. VLBW preterm infants diagnosed with CLD had lower gestational age compared to those without CLD. The parameters of lung function and indices of lung inhomogeneity were comparable between infants with and without CLD. Among all studied infants, FRC was positively related to the PMA and body length and tidal volume was positively correlated with the body weight and length. Moreover, FRC was positively correlated with the body length on measuring lung function in those with CLD.

## Figures and Tables

**Table 1:** Comparison of clinical variables between groups.

	CLD (*n* = 27)	No CLD (*n* = 18)	*p* value
Gestational age (weeks)	27.19 ± 1.98	29.61 ± 2.33	0.001
Birth weight (kg)	0.92 ± 0.21	1.24 ± 0.16	<0.001
Total days of respiratory support (days)	72.15 ± 21.79	29.61 ± 16.82	<0.001
Duration of intubation (days)	22.69 ± 26.8	2.17 ± 4.71	0.003
Male, *n* (%)	15 (55.6)	10 (55.6)	1
Prenatal steroid use, *n* (%)	12 (44.4)	6 (33.3)	0.543
RDS grade ≥3, *n* (%)	13 (48.1)	6 (33.3)	0.371
PDA, *n* (%)	20 (74.1)	5 (27.8)	0.005
Exogenous surfactant use, *n* (%)	10 (37)	2 (11.1)	0.086
Exogenous surfactant use ≥2 times, *n* (%)	5 (18.5)	1 (5.6)	0.377
Aminophylline use, *n* (%)	24 (88.9)	11 (61.1)	0.064

All continuous data are presented as mean ± SD. RDS: respiratory distress syndrome; PDA: patent ductus arteriosus.

**Table 2:** Comparison of pulmonary function parameters between groups.

	CLD (*n* = 27)	No CLD (*n* = 18)	*p* value
Postnatal age on checking lung function (days old)	93.30 ± 20.14	55.39 ± 20.16	<0.001
PMA on checking lung function (weeks)	41.33 ± 2.21	37.86 ± 2.42	<0.001
Body weight on checking lung function (kg)	2.73 ± 0.54	2.47 ± 0.61	0.010
Body length on checking lung function (cm)	47.06 ± 3.73	45.39 ± 4.00	0.121
*z*-score of body weight	−2.39 ± 1.25	−1.50 ± 1.25	0.018
*z*-score of body length	−2.40 ± 1.69	−1.48 ± 1.58	0.161
FRC (ml)	59.84 ± 22.54	56.24 ± 12.68	0.900
FRC (ml/kg)	19.29 ± 12.16	19.59 ± 11.21	0.757
FRC (ml/cm)	1.28 ± 0.47	1.24 ± 0.24	0.828
TV (ml)	23.68 ± 5.53	23.70 ± 3.91	0.828
TV (ml/kg)	8.86 ± 2.33	9.81 ± 1.40	0.050
MV (ml/kg)	1334.83 ± 682.92	1067.36 ± 529.21	0.285
RR (min)	78.31 ± 19.35	78.08 ± 16.07	0.959
*T*_I_ (s)	0.38 ± 0.09	0.39 ± 0.09	0.667
*T*_E_ (s)	0.44 ± 0.16	0.43 ± 0.10	0.823
*T*_I_/*T*_tot_	0.63 ± 0.19	0.60 ± 0.20	0.361
*T*_PTEF_ (s)	0.18 ± 0.13	0.19 ± 0.06	0.081
*T*_PTEF_/*T*_E (%)_	42.16 ± 18.21	47.12 ± 15.93	0.285
LCI	15.39 ± 4.28	15.97 ± 3.68	0.761
M1/M0	4.56 ± 1.26	5.44 ± 2.68	0.455
M2/M0	39.75 ± 21.05	43.95 ± 32.18	0.900

All continuous data are presented as mean ± SD. FRC: functional residual capacity; TV: tidal volume; *T*_I_: inspiratory time; *T*_E_: expiratory time; *T*_PTEF_: time to peak tidal expiratory flow; *T*_PTEF_/*T*_E_: the ratio of time to peak tidal expiratory flow over total expiratory time; M1/M0 and M2/M0: first and second moment ratios.

## Data Availability

The data used to support the findings of this study are included within the article.

## Abbreviations

CLD: Chronic lung disease
VLBW: Very low birth weight
PMA: Postmenstrual age
FRC: Functional residual capacity
RDS: Respiratory distress syndrome
PDA: Patent ductus arteriosus
TV: Tidal volume
MV: Minute volume
RR: Respiratory rate
*T*_I_: Total inspiratory time
*T*_E_: Total expiratory time
*T*_PTEF_: Time to peak tidal expiratory flow
LCI: Lung clearance index.
